# Mitochondrial Complex I Is an Essential Player in LPS-Induced Preconditioning in Differentiated PC12 Cells

**DOI:** 10.22037/ijpr.2019.1100711

**Published:** 2019

**Authors:** Nasim Manouchehri, Fariba Khodagholi, Leila Dargahi, Abolhassan Ahmadiani

**Affiliations:** a *Neuroscience Research Center, Shahid Beheshti University of Medical Sciences, Tehran, Iran. *; b *Neurobiology Research Center, Shahid Beheshti University of Medical Sciences, Tehran, Iran.*

**Keywords:** Mitochondrial complex I, Preconditioning, Neuroinflammation, Neuroprotection, Mitochondria

## Abstract

Preconditioning (PC) as a protective strategy against noxious insults can decline cell death and apoptosis. It has been approved that mitochondria play a key role in PC mechanism. The critical role of complex I (CI) in oxidative phosphorylation machinery and intracellular ROS production, particularly in the brain, accentuates its possible role in PC-induced neuroprotection. Here, differentiated PC12 cells were preconditioned with ultra-low dose LPS (ULD, 3 μg/mL) prior to exposure to high concentration of LPS (HD, 750 μg/mL). Our results showed that HD LPS treatment reduces cell viability and CI activity, and intensifies expression of cleaved caspase 3 compared to the control group. Intriguingly, PC induction resulted in enhancement of cell viability and CI activity and reduction of caspase3 cleavage compared to HD LPS group. In order to explore the role of CI in PC, we combined the ULD LPS with rotenone, a CI inhibitor. Following rotenone administration, cell viability significantly reduced while caspase3 cleavage increased compared to PC induction group. Taken together, cell survival and reduction of apoptosis followed by PC can be at least partially attributed to the preservation of mitochondrial CI function.

## Introduction

Lipopolysaccharides (LPS), known as endotoxins and lipoglycans, are the main part of the gram-negative bacteria outer membrane. LPS binds to the Toll-like receptors (TLRs) expressed by various types of cells in nervous system including neurons and glial cells and promotes the release of chemokines and pro-inflammatory factors such as tumor necrosis factor alpha (TNFα) and interlukin-1β (IL-1β) which ultimately induces neuroinflammation (1-3).

Preconditioning (PC) is a phenomenon in which different organs of the body like brain and heart will be protected against subsequent injurious damage by accommodating to low doses of harmful injuries. PC paradigms consist of low doses of endotoxins such as LPS, hypoxia, hypothermia, hyperthermia, anesthetics, 3-nitropropionic acid, *etc*. 

The PC mitigates neuroinflammation which results in neuroprotection. For example, LPS preconditioning plays a key role in reducing inflammation-induced apoptosis in spinal cord injury that consequently leads to neuroprotection (4). Although various mechanisms have been proposed for PC, the precise mechanism is unknown. Mitochondria as gate of cell death and survival, can play main role in PC mechanism (5). Leading role of mitochondria in the PC mechanism has been mostly considered in heart, brain, and neurodegenerative disorders (6, 7). Mitochondria produce adenosine tri-Phosphate (ATP) through electron transport chain (ETC) which are of particular importance to neurons (8). Mitochondrial impairment and oxidative phosphorylation (OXPHOS) disruption are unequivocal causes of neurodegenerative diseases such as Alzheimer′s disease, Parkinson′s disease and Huntington disease (9-17). Inflammation impairs mitochondrial function that leads to mitochondrial DNA (mtDNA) deficiency, mitochondrial respiratory chain dysfunction and eventually cell death (18-20). Mitochondrial respiratory chain is central source of ROS generation and its dysfunction results in increment of ROS production which consequently causes oxidative stress, a robust stimulus for promoting inflammation (21, 22). Therefore, mitochondrial dysfunction and excessive ROS production can lead to mitophagy and mitochondrial degradation (23). It has been recently shown that LPS can stimulate autophagy and mitophagy through oxidative stress induction. Whereas, LPS-induced tolerance could increase protein level of mitochondrial CI and mitochondrial complex IV (CIV) in THP-1 cells (24). The ETC, as a collection of proteins embedded in the inner mitochondrial membrane, is organized into five grand complexes named I to V. Passage of electrons from one complex of the transport chain to another lead to energy production. This process named proton gradient which is essential for ATP generation (25). The largest complex of ETC, complex I (NADH-ubiquinone oxidoreductase), oxidizes nicotinamide adenine dinucleotide (NADH) to NAD^+ ^and produces 2 electron that subsequently transfer to complex III (CIII). Moreover, the CI pumps proton from matrix to intermembrane space for generation of proton gradient (26). The CI is also the main origin of intracellular ROS, specifically in the brain’s mitochondria (27). Thus, it is not surprising that the CI inhibition can result in energy failure and increase ROS levels dramatically (8). Therefore, it has become evident that disturbance of mitochondrial CI function can cause the onset or progression of neurodegenerative disorders (13, 28-30). Given that the destructive effects of neuroinflammation on mitochondria may be attenuated by various mechanisms including generation of anti-inflammatory factors, preservation of mitochondrial integrity through mitophagy and production of new organelles by mitochondrial biogenesis (31, 32). However, the exact mechanism of these compensatory responses is unknown. Therefore, regarding the role of CI in OXPHOS machinery; here, we are going to reveal the role of mitochondrial CI in the mechanism underlying LPS-PC neuroprotection. 

## Experimental

*Cell culture*

This study received written approval from the Neuroscience Research Center Ethics Board (code IR.SBMU.PHNS.1394.49). PC12 cells (pheochromocytoma cells derived from the adrenal gland of Rattus norvegicus*)*, purchased from Pasteur Institute (Tehran, Iran), were grown in T-75 cm^2^ culture flasks in complete (DMEM) medium, containing 5% fetal bovine serum (FBS), 10% horse serum, and 1% antibiotic mixture comprising penicillin-streptomycin, at 37 °C with 5% CO_2_ and treated with nerve growth factor (NGF) (50 ng/mL) for 6 days (33). The cells were used when the density of the cell cultures reached 70–80%.

*Detection of the toxic dose of LPS*

Differentiated PC12 cells (1 × 10^4^/well), in 96-well plates, were treated with different LPS concentrations including 0.25, 0.5, 0.75, and 1 mg/mL, to find out the appropriate toxic dose of LPS mentioned as high dose (HD) later in this study. The cell survival rate was examined after 24 h, using MTT assay.

*Detection of appropriate time course of PC*

In order to find the best time for LPS-induced tolerance model, the differentiated PC12 cells were seeded at a density of 1 × 10^4^ cells/well in 96-well plates, and treated with ultra-low dose (ULD) of LPS (3 µg/mL). After 4, 6, 8, 12, and 24 h, the cells were exposed to HD LPS. After 24 h incubation, the cell viability was measured by the conventional MTT. 

Briefly, PC12 cells were seeded at a density of 1 × 10^6^ cells/mL in T-75 cm^2^ tissue culture flask and divided into six experimental groups: 1) Cells that received media as control group, 2) cells that received HD LPS (750 µg/mL) named as HD group, 3) cells treated by ULD LPS (3 µg/mL) for PC induction and after 12 h, exposed to HD LPS named as PC group, 4) cells that received rotenone (5 µM) as mitochondrial complex I inhibitor and named as R group (34), 5) cells that co-administrated with rotenone and HD LPS named as R+HD group, and 6) cells that were incubated with rotenone before PC induction and indicated as R+PC. 

The cells in all experimental groups, were harvested after 24 h and washed three times with phosphate buffered saline (PBS) for later tests.

*Measurement of cell viability*

Cell survival was assessed by the conventional MTT (3-[4, 5- dimethylthiazol-2-yl]-2,5-diphenyl tetrazolium bromide) reduction assay. Viable cells that contain intact mitochondria reduce MTT to dark purple formazan crystals. Then, formazan crystals were solubilized in Dimethyl sulfoxide (DMSO) and the absorbance was measured at 570 nm (35). Results are presented as the percentages of reduced MTT, assuming the absorbance of control cells as 100%.

*Preparation of total protein extracts*

To obtain total cell extracts, the treated cells were harvested, washed with ice-cold Phosphate buffered saline (PBS), and resuspended in lysis buffer. For western blot, the collected cells were lysed in RIPA buffer containing protease inhibitors and for complex activity assay; the proteins were extracted using the detergent provided by the assay kit manufacturer. After 30 min incubation on ice, cellular debris were removed by centrifugation at 13000 rpm at 4 °C for 25 min. Protein content was determined according to the Bradford’s method using bovine serum albumin (BSA) as a reference standard (36). 

*Western blot analysis*

Sixty microgram of total protein per sample were loaded on SDS-PAGE gel and transferred to polyvinylidene fluoride membrane (PVDF) (Millipore, Billerica, MA**)**. Subsequently, the membranes were blocked by non-fat dry milk-TBST solution (2%) and probed with primary antibodies (anti‐β‐actin as the housekeeping protein (1:1000) and Anti‐caspase 3 (1:1000)) overnight at 4 °C For immunodetection. After three times washing, the blots were incubated for 90 min at room temperature with horseradish peroxidase (HRP)-conjugated secondary antibody (1:3000). Immunoreactive polypeptides were detected by chemiluminescence using ECL kit reagents (Amersham Bioscience, Piscataway, NJ). Finally, the signals generated were quantified using the ImageJ software. 

*Determination of Mitochondrial complex I activity*

The cellular mitochondrial complex I activity was evaluated spectrophotometrically using Abcam′s microplate assay kit, according to the manufacturer′s instructions, which takes place by the oxidation of reduced NADH to NAD^+^ and the coincident reduction of a dye that results in enhanced absorbance at OD = 450 nm. Equal amounts of each sample were loaded onto the plate. Complex activity is expressed as the change in absorbance per minute per microgram protein loaded.

*Materials*

LPS from *Escherichia coli* 055:B5 (L2880) and rotenone (R8875) were purchased from Sigma Aldrich (St. Louis, MO, USA). Antibodies used against caspase-3 (9665) and β-actin (4970) were purchased from Cell Signaling Technology. The anti-rabbit horseradish peroxidase (HRP) linked secondary antibody (1:3000) (7074) was purchased from Cell Signaling Technology (Beverly, MA, USA). Mitochondrial complex I (ab109721) assay kit was purchased from Abcam (Cambridge, MA, UK).

*Statistical Analysis*

All data were analyzed using the one-way ANOVA followed by Tukey post-hoc test using GraphPad Prism software. In all analyses, *P* < 0.05 was considered statistically significant. All data were presented as mean ± SEM.

## Results

*The concentration of 0.75 mg/mL was selected as HD LPS*

**Figure 1:**
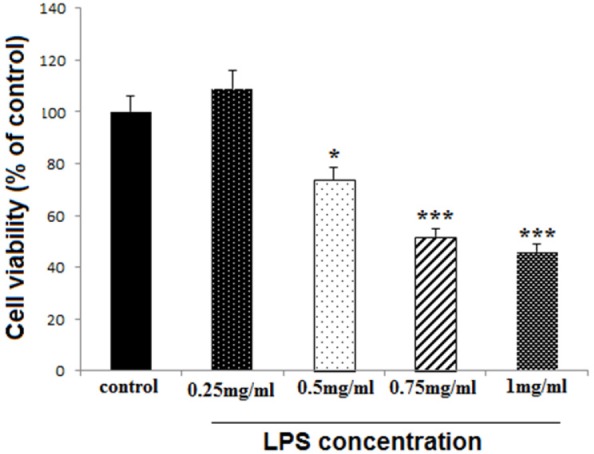
Effect of high dose LPS on cell viability in neural like cells. Cells (1 × 104/well) were treated with LPS at 0.25, 0.5, 0.75 and 1 mg/mL concentrations for finding toxic dose, and then, cell viability was evaluated MTT assay. LPS reduced cell viability at 0.5, 0.75 and 1 mg/mL concentrations, while the lower concentration did not change cell viability. Data are shown as mean ± SEM. Treatments were repeated 3 times and there were 4 wells in each repeat. *P < 0.05, ***P < 0.001 *vs. *Ctrl

LPS reduced cell viability at 0.5, 0.75 and 1 mg/mL concentrations (*P < *0.05, *P < *0.001 and *P < *0.001, respectively), while the lower concentration (0.25 mg/mL) did not change cell viability (Figure 1). The concentration of 0.75 mg/mL was selected for later experiments and considered as HD LPS.

*The time course of 12 h was selected for PC induction*

**Figure 2:**
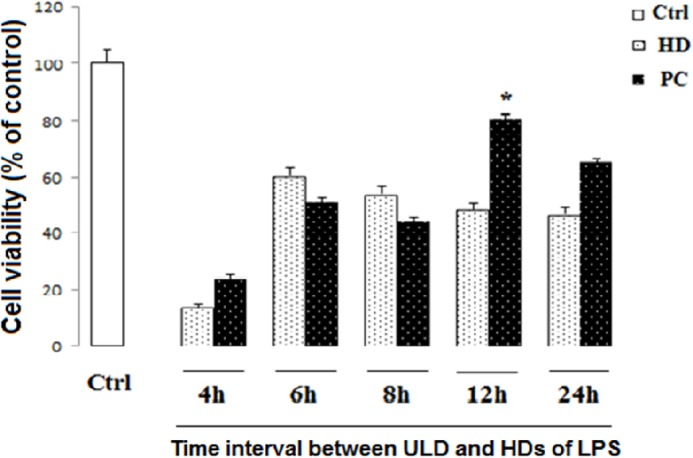
Effect of PC and time of incubation on cell viability in neural like cells. Cells (1 × 104/well) were treated with HD LPS and ultra-low dose (ULD) LPS for PC induction for 4, 6, 8, 12, and 24 h, and then, cell viability was measured using MTT test. Cell viability reduced following HD LPS. PC increased cell viability following 12 h interval between HD and ULD, while other intervals did not change cell viability. Data are shown as mean ± SEM. Treatments were repeated 3 times and there were 4 wells in each repeat

PC12 cells treated with ULD LPS were exposed to HD LPS as the toxic dose after 4, 6, 8, 12, and 24 h. Our data showed that pretreatment by ULD LPS 12 h before the HD exposure leads to preservation of cell viability compared to HD LPS (*P < *0.05). This is while, as shown in Figure 2, intervals of 4, 6 and 8 h between ULD and HD LPS didn’t show any significant difference in cell survival in comparison to HD LPS treatment alone. On the other side, because there was statistically no significant discrepancy between PC induction and ctrl group in pretreatment by ULD LPS 24 h before the HD exposure, therefore 24 h interval between ULD and HD could lead to conservation of cell survival. 

*Complex I activity was preserved by PC induction*

**Figure 3:**
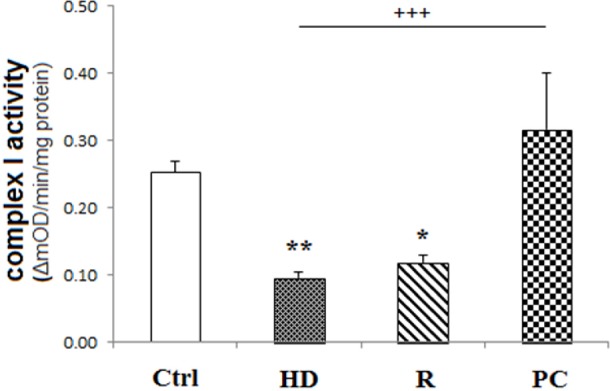
High dose LPS reduced the activity of complex I. HD LPS reduced complex I activity while PC preserved this activity. Data are shown as mean ± SEM. Treatments were repeated 3 times. *P < 0.05, **P < 0.01 *vs. *Ctrl, +++*P < *0.001 *vs. *HD

The activity of mitochondrial CI activity was expressed as the change in absorbance per minute per microgram protein loaded. As shown in Figure 3, Complex I activity reduced in HD treated cells (*P < *0.01). But, PC induction preserved complex I activity compared to HD group (*P < *0.001). Rotenone, as CI inhibitor, decreased CI activity (*P < *0.05).

Ctrl: control; HD: high dose lipopolysaccharides (LPS).

*P < 0.05 *vs. *Ctrl.

Ctrl: control; HD: high dose lipopolysaccharides (LPS); PC: preconditioning.

Ctrl: control; HD: high dose lipopolysaccharides (LPS); PC: preconditioning; R: Rotenone.

**Figure 4:**
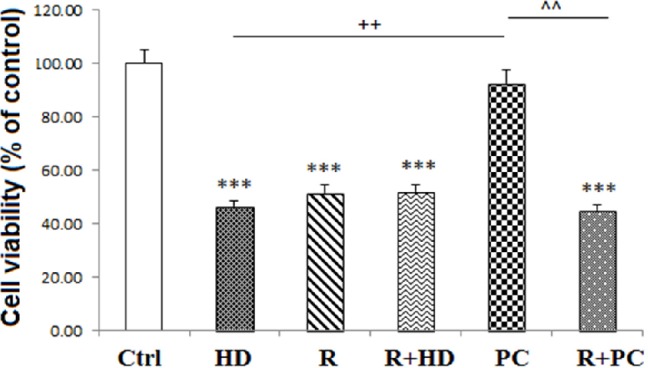
Effect of inhibition of complex I and PC on neural like cells measured using MTT test. Inhibition of complex I attenuated the protection effect of PC on cell viability. Data are shown as mean ± SEM. Treatments were repeated 3 times and there were 4 wells in each repeat. ***P < 0.001 *vs. *Ctrl, ++*P < *0.01 *vs. *HD, ^^*P < *0.01 *vs. *PC. Ctrl: Control; HD: high dose lipopolysaccharide (LPS); PC: preconditioning; R: Rotenone

**Figure 5:**
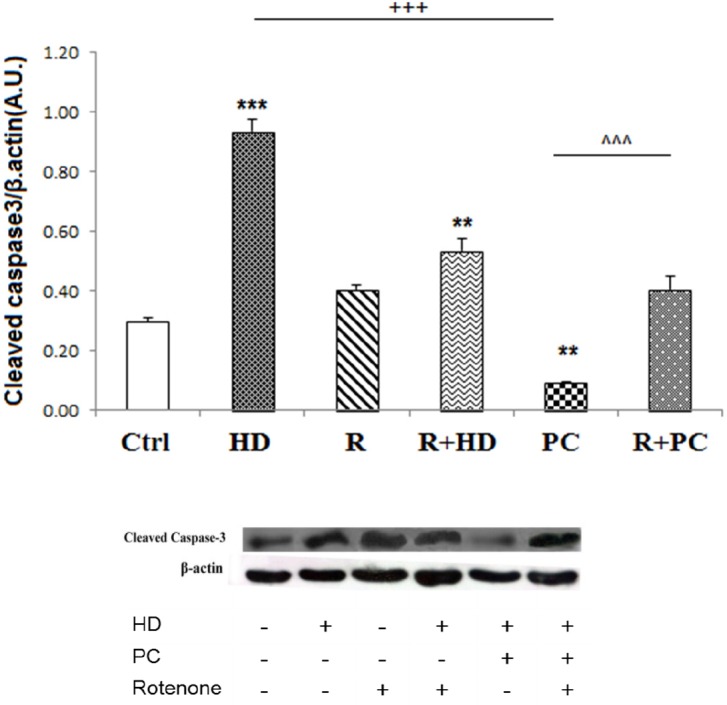
The role of complex I in protective effect of PC against caspase 3 cleavage induced by high dose LPS. Inhibition of complex I by rotenone increased caspase 3 cleavage. PC prevented the activation of apoptotic pathways in. response to high dose LPS. Data are shown as mean ± SEM. Treatments were repeated 3 times. **P < 0.01; ***P < 0.001 *vs. *Ctrl, +++*P < *0.001 *vs. *HD, ^^^*P < *0.001 *vs. *PC. Ctrl: control; HD: high dose lipopolysaccharides (LPS); PC: preconditioning; R: Rotenone

*Inhibition of mitochondrial complex I, abolished the protective effects of PC on cell survival*

As shown in Figure 4, suppression of mitochondrial CI activity by rotenone and co-treatment of cells with both rotenone and HD LPS significantly reduced cell viability (*P < *0.001 and *P < *0.001, respectively). Interestingly, co-administration of rotenone with PC induction decreased cell viability in neural like cells in comparison with PC group (*P < *0.01).

*The protective effect of PC against caspase 3 cleavage was attenuated by the inhibition of mitochondrial complex I*

The protein levels of cleaved caspase-3 were measured as a pro-apoptotic factor. Consistent with cell survival data, the western blot data showed that HD LPS increased cleaved caspase3 expression (*P < *0.001). While rotenone treatment alone did not enhance caspase3 cleavage, co-treatment of rotenone with HD increased the cleaved caspase3 compared to control group (*P < *0.01). Induction of PC significantly decreased expression of cleaved caspase3 compared to the HD (*P < *0.001). Also, it was observed that co-administration of rotenone with PC induction enhanced expression of cleaved caspase3 compared to the PC induction (Figure 5).

## Discussion

Regarding the role of mitochondria in pathogenesis of neurodegenerative disorders such as Alzheimer′s disease, Parkinson′s disease, and neuroinflammatory diseases like multiple sclerosis (MS) and indispensable protective role of PC in neural damage, in the present study, the main question is that whether mitochondrial CI, that plays a crucial role in ATP generation has any possible role in the PC mechanism. To get insight into the link between CI function and PC mechanism, we examined cell viability, caspase3 cleavage, and the activity of mitochondrial CI. The main findings from this in vitro study were the following: i), 750 µg/mL concentration is the toxic dose of LPS, ii) The most appropriate time for the PC induction is 12 h interval between ULD LPS and HD LPS, iii) Reduction of caspase3 cleavage and increment of cell survival occur following LPS-induced PC, iv) The activity of CI was preserved by LPS-induced PC, and v) Blockage of the CI activity by rotenone suppressed the protective effects of the PC.

Our results showed that 750 µg/mL concentration is the toxic dose of LPS and the most appropriate time for PC induction is 12 h interval between ULD LPS (for inducing protection) and HD LPS (for noxious insult). In fact, 12 h preconditioning interval increased cell survival from 48% to 81%. On the other hand, since there is no statistically significant difference between PC induction and control group in pretreatment by ULD LPS 24 h before the HD exposure, 24 h interval between ULD and HD can also result in preservation of cell survival. Given the higher percentage of cell survival in 12 h interval, it seems that the PC induces the most effective protection in this time. Consequently, 12 h interval between PC induction and HD LPS exposure was selected as the most appropriate time for PC induction.

Preconditioning with H_2_O_2 _for 90 min in PC12 cells, protect the cells against 24 h later exposure to the higher concentrations of H_2_O_2_ (37, 38). Meloni and colleagues used erythropoietin (EPO) for PC induction at 8, 12, and 24 h before *in-vitro* model of ischemia and indicated that EPO preconditioning in neurons and PC12 cells protects them against harmful consequences of ischemia. They also reported that cell viability enhances from 26% to 61% in 8 h PC interval while increase of cell survival up to 89% has been observed in 12- and 24 h PC intervals (39). It has been also demonstrated that 24 h interval between low dose LPS PC and surgery ameliorates the cognitive damage and prohibits neuroinflammation in hippocampus following surgery (40). 

LPS has been used frequently to induce preconditioning in several studies. For example, it has been shown that pre-treatment with a single intraperitoneal injection of LPS, 7 days prior to traumatic brain injury, causes a significant neuroprotection in the brain of animals against axonal injury (41). In the other study, preconditioning with LPS 72 h before middle cerebral artery occlusion and 4 days before hemorrhagic shock have proved promising results (42-44). Hence, the neuroprotective effects of LPS or any other preconditioning-inducing agents mainly depend on the time of administration. In general, the best time interval for the applying preconditioning depends on i) the type of paradigms such as H_2_O_2_, EPO and LPS, ii) the pathological context, and iii) the study platform, *in-vivo/in-vitro*.

Given that the MTT test relies on the tetrazolium dye reduction by the mitochondrial dehydrogenases, thus, caspase 3 cleavage as a mitochondrial dependent cell death factor was also investigated. Our findings showed that although HD LPS reduced viable cells and increased caspase 3 cleavage, PC preserved cell survival and significantly attenuated cleaved caspase 3 expression. In agreement with these findings, it has been reported that hyperbaric oxygen PC suppresses pro-apoptotic factors such as caspase3, 7, 8, and 12 following spinal cord injury (45). It has been also indicated that oxygen and glucose deprivation PC significantly attenuate apoptotic cell death in PC12 cells in a cellular model of ischemia (46). 

In the next step of study, our data also showed that HD LPS reduced CI activity. Consistent with our results, Callahan and Supinski have reported that the down-regulattion of mitochondrial CI, II, and IV occur at the transcription and translation levels within 24 h after exposure to toxic doses of LPS in sepsis model of rats (47). Therefore, other evidence suggests that dysfunction of mitochondrial complexes occurs following neuroinflammation in striatum, substantia nigra, and midbrain in animal models of Parkinson′s disease (48-50). Indeed, augmented levels of inflammatory mediators can influence mitochondrial function (51, 52). For example, TNFα and IL-1β reduce mitochondrial membrane potential (MMP), ATP generation, and mitochondrial CI activity (53, 54). Zhang and colleagues indicated that LPS PC may notably reduce cognitive damage and surgeryinduced neuroinflammation in aging mice (40). It has been also shown that LPS PC can protect dopaminergic neurons against neuroinflammation in rat midbrain (55). Our findings demonstrated that the activity of CI was preserved by LPS-induced PC; this observation is supported by other studies emphasizing the protective effects of PC on mitochondrial structure and function (4, 40, 41, 56 and 57). In a recent study it has been shown that isoflurane PC increases the expression of anti-apoptotic factors including Bcl-XL and Bcl-2, decreases apoptosis, preserves mitochondrial complexes activity and restricts ROS production in neurons (58). It has been demonstrated that complexes I-IV activity preserves following of ischemic PC (IPC) induction (5, 59 and 60). Dave and colleagues showed that there is no significant difference in activity of hippocampal mitochondrial complexes I to IV and oxygen consumption rates between ischemic PC and sham groups (61). In another study, it has been shown that LPS-PC result in mitochondrial biogenesis through the increase of ATP generation, overexpression of mitochondrial protein levels, and enhancement of respiration capacity *in-vitro* model of ischemia (62). 

In the present study, to signify the role of mitochondrial CI in PC-induced protection, we used rotenone as a specific CI inhibitor and the results showed that LPS-induced PC increases cell survival, reduces caspase3 cleavage and stimulates CI activity compared to the cells that received co-administration of rotenone and PC. Rotenone as a robust inhibitor of CI disturbs electron transfer in the ETC, decreases ATP production and enhances ROS generation that ultimately result in oxidative stress and apoptotic cell death (63). Heinz *et al.* reported that rotenone declines CI activity in isolated liver mitochondria in rats (64). No significant difference between rotenone group and co-treatment of rotenone and PC induction suggests that inhibition of CI can result in reduction of cell viability and prevention of PC-induced neuroprotection. In the present study our data demonstrated that LPS PC protects neural like cells against inflammatory agents and preserves CI activity. Moreover, blockage of CI activity by rotenone suppressed the protective effects of PC which highlights the major role of mitochondria, specifically CI activity, in the mechanism of LPS-induced PC. Decline of cleaved caspase3 protein levels and increment of cell survival following LPS-induced PC signifies the PC as a protective approach which can be assumed as a novel therapeutic target for prevention and/or treatment of neurodegenerative disorders. 

## Conclusion

The protective effects of low concentrations of PC compared to the toxic effects of higher concentrations of LPS can be attributed to the preservation of mitochondrial function, particularly the CI activity. 
